# Cyclin-dependent kinase complexes in developing maize endosperm: evidence for differential expression and functional specialization

**DOI:** 10.1007/s00425-013-1990-1

**Published:** 2013-11-16

**Authors:** Ricardo A. Dante, Paolo A. Sabelli, Hong N. Nguyen, João T. Leiva-Neto, Yumin Tao, Keith S. Lowe, George J. Hoerster, William J. Gordon-Kamm, Rudolf Jung, Brian A. Larkins

**Affiliations:** 1 School of Plant Sciences, University of Arizona, 303 Forbes, Tucson, AZ 85721 USA; 2 Pioneer Hi-Bred International, Inc., Johnston, IO 50131 USA; 3 Present Address: Embrapa Agricultural Informatics, Av. André Tosello 209, Campinas, SP 13083-886 Brazil

**Keywords:** Cell division, Cell expansion, Endoreduplication, Proteasome, *Zea*

## Abstract

Endosperm development in maize (*Zea mays* L.) and related cereals comprises a cell proliferation stage followed by a period of rapid growth coupled to endoreduplication. Regulation of the cell cycle in developing endosperm is poorly understood. We have characterized various subunits of cyclin-dependent kinase (CDK) complexes, master cell cycle regulators in all eukaryotes. A-, B-, and D-type cyclins as well as A- and B-type cyclin-dependent kinases were characterized with respect to their RNA and protein expression profiles. Two main patterns were identified: one showing expression throughout endosperm development, and another characterized by a sharp down-regulation with the onset of endoreduplication. Cyclin CYCB1;3 and CYCD2;1 proteins were distributed in the cytoplasm and nucleus of cells throughout the endosperm, while cyclin CYCD5 protein was localized in the cytoplasm of peripheral cells. CDKB1;1 expression was strongly associated with cell proliferation. Expression and cyclin-binding patterns suggested that CDKA;1 and CDKA;3 are at least partially redundant. The kinase activity associated with the cyclin CYCA1 was highest during the mitotic stage of development, while that associated with CYCB1;3, CYCD2;1 and CYCD5 peaked at the mitosis-to-endoreduplication transition. A-, B- and D-type cyclins were more resistant to proteasome-dependent degradation in endoreduplicating than in mitotic endosperm extracts. These results indicated that endosperm development is characterized by differential expression and activity of specific cyclins and CDKs, and suggested that endoreduplication is associated with reduced cyclin proteolysis via the ubiquitin–proteasome pathway.

**Electronic supplementary material:**

The online version of this article (doi:10.1007/s00425-013-1990-1) contains supplementary material, which is available to authorized users.

## Introduction

Maize (*Zea mays* L.) endosperm development is characterized by three distinct and successive types of cell cycle: acytokinetic mitosis, which produces a syncytium; mitotic cell division; and endoreduplication (reviewed by Sabelli and Larkins 2009a). Following cellularization of the syncytium around three days after pollination (DAP), the endosperm grows mostly by mitotic cell divisions, which occur most frequently at 8–10 DAP, and subsequently cease in central cells but persist at low frequency in peripheral cells through later developmental stages (Kiesselbach 1949; Kowles and Phillips 1985; Lur and Setter 1993). Also by 8–10 DAP, initiating in central regions of the endosperm and extending toward its periphery, cells gradually and asynchronously cease dividing and engage in the endoreduplication cell cycle, which is characterized by repeated rounds of DNA replication without intervening sister chromatid segregation and cytokinesis (Edgar and Orr-Weaver 2001). This cell cycle variant results in highly polyploid cells with multiple, apparently uniform, copies of chromosomes. Ploidy levels and cell sizes are highly correlated in numerous cell types and, accordingly, the spatiotemporal pattern of transition from mitosis to endoreduplication creates a gradient of nuclear ploidy and cell size in the endosperm. Small, non-endoreduplicated cells are located at the periphery of this tissue and increasingly large and endoreduplicated cells towards its center. Endoreduplicated cells account for the major fraction of endosperm volume (Vilhar et al. 2002).

In eukaryotes, the cell cycle is controlled by the periodic activity of heterodimeric complexes of threonine/serine cyclin-dependent kinases (CDKs) and their activating cyclin subunits. It has been established that distinct CDKs and their cyclin partners control progression through the cell cycle phases in plants (reviewed by Inzé and De Veylder 2006). Among the major types of CDKs, members of the A-type contain a PSTAIRE motif in the cyclin-interacting α-helix and function during S-phase and at the G1/S and G2/M transitions. The plant-specific B-type CDKs, in which the PSTAIRE motif is replaced by PPTALRE (B1-subtype) or PPTTLRE (B2-subtype), function primarily at the G2/M transition. Plants possess a large number of cyclins, and typically utilize D-type cyclins to control the G1/S transition, A-type cyclins to control S-phase and the G2/M transition, and B-type cyclins to control G2/M and intra-mitotic transitions. The primary mechanisms that regulate the activity of CDK complexes include binding of non-catalytic CDK-specific inhibitors (CKIs), the phosphorylation status of the CDK subunit, and cyclin synthesis and proteolysis, the latter of which is mediated by the ubiquitin/26S proteasome pathway. The anaphase promoting complex/cyclosome (APC/C), Skp1/Cullin/F-box complex, and Cullin-RING ubiquitin Ligases are the major multimeric E3 ubiquitin-protein ligases that target cyclins and other cell cycle regulators to the proteasome and thus promote cell cycle progression (Peters 2006; Marrocco et al. 2010; Mocciaro and Rape 2012; Heyman and De Veylder 2012). During the late stages of mitosis and most of the G1 phase, CDK activity is typically reduced by the proteolysis of A- and B-type cyclins via the APC/C (Peters 2006). In higher eukaryotes, members of the retinoblastoma-related (RBR) family of proteins typically repress the G1/S transition by inhibiting the activity of heterodimeric E2F/DP transcription factors, which control the expression of many genes required for S-phase and cell cycle progression (reviewed by Sabelli and Larkins 2009b). Transitioning into S-phase involves the phosphorylation and inhibition of RBR proteins by CDK complexes, which relieves the block on E2F/DP-dependent gene expression.

The cell cycle mechanisms associated with endoreduplication in various plant cell types have been discussed in detail (Chevalier et al. 2011; De Veylder et al. 2011; Sabelli 2012). In particular, the APC/C plays important roles in this cell cycle in many cell types by degrading mitotic cyclins and thus reducing corresponding CDK activities. However, while many of these mechanisms appear to be common, their individual contributions may be cell type-specific (Roodbarkelari et al. 2010; De Veylder et al. 2011). Endoreduplication in maize endosperm appears to involve oscillation of S-phase CDK activity coupled with down-regulation of M-phase CDK activity (Grafi and Larkins 1995). A number of observations are consistent with this view. Endoreduplication is reduced by over-expression of a catalytically inactive form of an A-type CDK (Leiva-Neto et al. 2004) and stimulated by decreased *RBR1* activity and consequent up-regulation of E2F/DP-dependent gene expression (Sabelli et al. 2013). In addition, endoreduplication in maize endosperm correlates with the expression of cyclin-dependent kinase inhibitors (CKIs) of the Inhibitor of Cdc2 Kinase/Kip-Related Protein (ICK/KRP)-type (Coelho et al. 2005), the contrasting expression and roles of different RBR proteins (Sabelli et al. 2005, 2009, 2013), and the reduced expression of *CYCB1;3* transcripts (Sun et al. 1999).

Despite growing knowledge regarding cell cycle mechanisms governing maize endosperm development, the identity, expression and regulation of cyclins and CDKs and their roles in the mitotic and endoreduplication cell cycles in this tissue are poorly understood. To date, only expression of the B1-type cyclin, CYCB1;3, has been thoroughly examined at the RNA level (Sun et al. 1997, 1999), and functional studies have been limited to the role of CDKA;1 during endoreduplication (Leiva-Neto et al. 2004). Here, we characterize various types of CDKs and cyclins during maize endosperm development with respect to their expression patterns as well as their associated kinase activities. This investigation revealed contrasting features of cell cycle control in maize compared to other model species. Analysis of previously uncharacterized CDKs suggested the existence of redundancy among A-type CDKs and a specialized role for a B1-type CDK at stimulating cell proliferation. In addition, a discrepancy between the levels of CYCB1;3 transcript and protein, along with the persistence of CYCB1;3 protein in endoreduplicated cells was discovered, which prompted an analysis of proteasome-dependent proteolysis of cyclins. These results revealed specificities in the spatiotemporal expression of different cyclins and CDKs and in the activity of cyclin/CDK complexes during endosperm development, and suggested that the transition from mitotic to endoreduplication cycles and its concomitant cell expansion are associated with reduced proteasome-dependent proteolysis of several types of cyclins.

## Materials and methods

### Database search and sequence analysis

Maize cyclin and CDK sequences were identified via BLAST (Altschul et al. 1990) searches of Pioneer Hi-Bred’s maize EST and GenBank databases using sequences of D-, A- and B-type cyclins, and A- and B-type CDKs from various plant species as queries. Subsequent searches were made in the Maize Genome Sequencing Project database (http://www.maizesequence.org). Analyses of maize transcriptome datasets were made with qTeller (http://qteller.com/qteller3/) and Maize eFP Browser (Patel et al. 2012) (http://bar.utoronto.ca/efp_maize/cgi-bin/efpWeb.cgi). Predictions of subcellular localization were made with WoLF PSORT (Horton et al. 2007) (http://wolfpsort.org/) and TargetP 1.1 (Emanuelsson et al. 2007) (http://www.cbs.dtu.dk/services/TargetP/).

### Plant materials

Maize (*Zea mays* L.) plants of the inbred line B73 (obtained from Maize Genetics Cooperation Stock Center, University of Illinois at Urbana-Champaign, IL, USA) and the transgenic line *RBR1DS1* (Sabelli et al. 2013) were grown in the field or in a greenhouse and hand-pollinated. Developing kernels were harvested at different stages and either frozen in liquid N_2_ and stored at −80 °C for molecular analysis, immediately analyzed by flow cytometry, or fixed for immunohistochemistry. RNA and protein analyses were performed on manually dissected frozen endosperm.

### RT-PCR

Total RNA was extracted with an Absolutely RNA RT-PCR Miniprep Kit (Stratagene, La Jolla, CA, USA). cDNA was synthesized from 25 ng of total RNA and end-point ^32^P RT-PCR was performed using the Titan one-tube RT-PCR kit (Roche, Hamburg, Germany) as described by Sabelli et al. (2005) with the primers listed in Online resource 1. Annealing steps were at 65 °C. Amplification was performed with 0.7 μl of 10 mCi/ml (370 MBq/ml) α[^32^P] dCTP (NEN, Boston, MA, USA); typically, 24–27 cycles were performed for amplification of cyclin and CDK RNA and 17 cycles for actin RNA. Using these conditions, amplification was within the exponential range. Reaction products were separated by electrophoresis in 4 % polyacrylamide/TBE gels (90 mM Tris, 64.6 mM boric acid pH 8.3, 2.5 mM EDTA), dried, and exposed to a Phosphorscreen (Molecular Dynamics, Sunnyvale, CA, USA), which was scanned with a Storm 860 PhosphorImager (Molecular Dynamics). Quantitation was performed with ImageQuant software (Amersham, Piscataway, NJ, USA).

Real-time quantitative RT-PCR analysis of *CDKB1;1* expression in wild-type and *RBR1DS1* endosperms was performed essentially as described by Sabelli et al. (2013). Briefly, segregating wild-type and transgenic *RBR1DS1* kernels were genotyped by PCR, and three pools containing RNA extracted from four endosperms were made for each genotype. One and a half μg RNA was reverse-transcribed into first strand cDNA using SuperScript VILO cDNA Synthesis Kit (Invitrogen, Carlsbad, CA, USA). cDNA samples were diluted 20-fold and 2 μl used as template in PCR reactions with Power SYBR Green Master Mix (Applied Biosystems). The final primer concentration was 300 nM, and reactions were run in an Applied Biosystems 7300 Real-Time PCR instrument. Data were analyzed with 7300 System SDS software (Applied Biosystems). RNA expression fold-changes were normalized relative to those of actin transcript and calculated according to the ΔΔCt formula: fold change = 2^− [(Gene × Ct − actin Ct) (in mutant)] − [(gene × Ct − actin Ct) (in control)]^.

### Expression and purification of protein fusions in *E. coli*

Sequences encoding selected regions of cyclins (amino acid residues 1–243 of CYCA1;1, 1–206 of CYCB1;3, 263–358 of CYCD2;1 and 243–349 of CYCD5;1) and full-length CDKA;1 and CDKB1;1 were amplified from cDNA clones with *Pfu* DNA polymerase (Stratagene, San Diego, CA, USA). Amplified fragments were cloned into pGEX6P-3 or pGEX4T-3 expression vectors (Amersham) and the nucleotide sequences verified by DNA sequencing. Glutathione-*S*-transferase (GST) fusions were expressed in *E. coli* BL21(DE3) Codonplus RIL (Stratagene) by induction of cultures with 0.1 mM (CDKs) or 1 mM (cyclins) isopropyl-β-d-thiogalactopyranoside at 30 °C (CDKs) or 37 °C (cyclins) for 3 h. After collecting the cells, they were lysed for 30 min on ice in extraction buffer (50 mM Tris–HCl pH 7.5, 100 mM NaCl, 1 mM EDTA, and 1 mM DTT) supplemented with 1 mM phenylmethylsulfonyl fluoride, 1 % Triton X-100, and 1 mg/ml lysozyme, followed by sonication. Lysates were cleared by centrifugation at 15,000*g* for 15 min, and incubated with glutathione-agarose beads (Sigma, St. Louis, MO, USA) at 4 °C. Beads were washed extensively and GST fusions eluted with 10 mM glutathione. For GST-CDKB1;1 purification, cells were lysed in 2 M urea, 50 mM Tris–HCl, pH 7.5, followed by extraction from inclusion bodies with 8 M urea, 50 mM Tris–HCl, pH 7.5.

### Antibodies

Purified GST fusions were separated by SDS-PAGE; gels were stained with 0.05 % Coomassie Blue R250 in distilled H_2_O and gel regions containing antigens were excised. New Zealand White rabbits were injected with 200 μg of protein four times at Strategic BioSolutions (Newark, DE, USA). For affinity purification of antibodies, an IgG-enriched fraction was obtained from crude antisera by precipitation at 50 % NH_3_SO_4_ saturation and depleted of GST antibodies by incubation overnight at 4 °C with GST covalently linked onto Reacti-Gel 6X agarose beads (Pierce, Rockford, IL, USA). The supernatant was incubated overnight at 4 °C with the corresponding cyclin or CDK–GST fusion linked to Reacti-Gel 6X. Antibody-adsorbed beads were washed extensively with 50 mM Tris–HCl pH 7.5, and antibodies eluted with 100 mM NaCl, 100 mM glycine, pH 2.4. Collected fractions were neutralized, pooled and concentrated with Centricon-10 spin cartridges (Millipore, Billerica, MA, USA), followed by exchange with phosphate buffered saline (PBS), pH 7.2. The specificity of cyclin and CDK antibodies was determined by immunoblot analysis of proteins expressed in Drosophila S2 cells, endosperm extracts, and, limited to cyclins, in vitro-translated proteins (Online Supporting Information). Pre-immune sera from immunized rabbits were subjected to the same affinity purification procedure and used as controls at the same dilution as their immune counterparts. Mouse monoclonal anti-actin (N350) and anti-PSTAIR (P7962) antibodies were purchased from Amersham and Sigma, respectively.

### In vitro transcription/translation of cyclin and other gene coding sequences

Coupled transcription/translation of full-length cyclins, 27-kDa γ-zein, and firefly luciferase was performed with TNT T7 Quick for PCR DNA (Promega, Madison, WI, USA). Primer design and reactions were performed according to the manufacturer’s instructions. Synthesis of radio-labeled proteins for stability assays was performed with [^35^S]-l-methionine (Amersham) at a final concentration of 0.8 μCi/μl (29.6 Bq/μl).

### Extraction of protein from maize endosperm

Dissected endosperm was homogenized in three volumes of ice-cold buffer either with a Polytron (Kinematica AG, Cincinnati, OH, USA) or in conical microcentrifuge tubes with plastic pestles. For immunoblotting and immunoprecipitation assays, endosperm was homogenized in NETT buffer (20 mM Tris–HCl pH 7.5, 100 mM NaCl, 5 mM EDTA, 0.5 % Triton X-100, and 1 mM DTT) supplemented with 1 mM β-glycerophosphate, 1 mM NaF, 1 mM Na_2_VO_4_, and Complete EDTA-free Protease Inhibitor Cocktail (Roche). Homogenates were cleared by centrifugation at 15,000*g* for 15 min at 4 °C. For protein stability assays, dissected endosperms were homogenized in buffer (50 mM Tris–HCl pH 7.5, 100 mM NaCl, 5 mM MgCl_2_, 1 mM DTT, 2 mM ATP, and 0.5 % Triton X-100) and cleared by centrifugation at 500*g* for 10 min at 4 °C. Supernatants were either used immediately or frozen in liquid N_2_ for subsequent use. Protein concentration was determined by the Bradford assay (Protein Assay kit, Bio-Rad, Hercules, CA, USA), using bovine serum albumin (BSA) (Pierce) as standard.

### Immunoblotting of maize endosperm extracts

Protein extracts from endosperm (50 μg per lane for detection of cyclins and CDKs, and 10 μg for actin) were separated by 10 or 12.5 % SDS-PAGE and blotted onto nitrocellulose membranes (Protran BA 85, Schleicher and Schuell, Dassel, Germany) in a tank transfer system (Mini Trans-Blot Cell, Bio-Rad) at 200 Vh in TGM buffer (25 mM Tris, 192 mM glycine, 20 % methanol, pH 8.3). Membranes were blocked with 5 % fat-free milk solids in TBST (20 mM Tris–HCl pH 7.5; 150 mM NaCl; and 0.05 % Tween-20) and incubated with antibodies (100 ng/ml anti-cyclins and anti-CDKs, 300 ng/ml anti-actin) in 5 % powdered milk in TBST overnight at 4 °C. Membranes were washed with TBST for 10 min three times and incubated with a 1:10,000 dilution in TBST of anti-rabbit (Pierce) or anti-mouse (Sigma) IgG horseradish peroxidase-conjugate, and developed with SuperSignal West Pico Chemiluminescent Substrate System (Pierce) followed by exposure to radiographic film.

### Immunohistochemistry

Developing maize kernels were analyzed using Steedman’s wax-embedded sections as described (Brown and Lemmon 1995; Nguyen et al. 2001), with minor modifications. Medial longitudinal 1–2 mm thick sections from freshly harvested kernels were fixed in 4 % formaldehyde in PHEM-DMSO buffer (50 mM Pipes pH 6.8, 5 mM EGTA, 1 mM MgSO_4_·7H_2_O, and 1 % DMSO) overnight at 4 °C. Sections were washed thoroughly in PHEM-DMSO buffer, dehydrated in a graded ethanol series, and slowly infiltrated with Steedman’s wax. Embedded kernels cut in 10 μm sections were adhered to coverslips coated with Mayer’s egg albumin adhesive and de-waxed with absolute ethanol. The tissue was permeabilized with a cocktail of 1 % each of cellulase, BSA, and Triton X-100 in PBS, pH 7.0, for 30 min at room temperature. Antibodies were diluted at 0.5–1 μg/ml in 0.25 % BSA in PBS, and incubated with the sections at 37 °C for 1 h. This was followed by incubation in distilled deionized H_2_O for 1 h at 37 °C with appropriate minimal cross-reactivity secondary antibodies conjugated to either rhodamine red or fluorescein (Jackson Immunoresearch, West Grove, PA, USA), diluted 1:200. Coverslips containing sections were mounted in Prolong AntiFade (Molecular Probes, Eugene, OR, USA) and allowed to dry in the dark overnight. Counterstaining of nuclei was made with the double-stranded DNA-preferred dye TO-PRO 3-iodide (Molecular Probes). Images were collected on a Bio-Rad MRC 1024 confocal laser scanning microscope and processed with NIH Image software (http://rsb.info.nih.gov/nih-image/) and Adobe Photoshop (San Jose, CA, USA).

### Gene expression in Drosophila S2 cell cultures

Full-length coding sequences were amplified from cDNA clones with Pfu DNA polymerase, cloned into pHSKSMCS (a gift from Thomas Bunch, University of Arizona), and verified by sequencing. pHSKSMCS contains the *D. melanogaster* *HSP70* promoter, the pBluescript multi-cloning site (at *Kpn*I-*Sac*I orientation), and an α-tubulin terminator on a pUC18 backbone. S2 cells (Schneider 1972) were cultured in Shields and Sang M3 medium (Sigma) supplemented with 10 % fetal calf serum (Sigma), 100 units ml^−1^ penicillin and 100 μg ml^−1^ streptomycin, at 25 °C. Approximately, 2 × 10^6^ cells were resuspended in 1 ml of M3 medium containing a mixture of 10 μl of Cellfectin (Invitrogen) and equal amounts of DNA for each construct (up to 2 μg combined). After a 6-h incubation at 25 °C, 0.5 ml of medium supplemented with 30 % fetal calf serum was added to the cell cultures. Expression was induced 24–36 h after transfection by heat shock (37 °C, 1 h). Cells were allowed to recover at 25 °C for 2 h, harvested by centrifugation, and frozen in liquid N_2_. Cells were resuspended in ice-chilled NETT buffer supplemented with 1 mM β-glycerophosphate, 1 mM NaF, 1 mM Na_2_VO_4_, and Complete EDTA-free Protease Inhibitor Cocktail, and sonicated for 5 s. Lysates were cleared by centrifugation at 15,000*g* for 15 min at 4 °C and used in immunoblots or immunoprecipitation assays.

### Immunoprecipitation of cyclins and kinase activity assay

Forty microliters of 50 % protein A-agarose (Sigma) slurry was added to endosperm soluble protein extract (2 mg) and incubated for 1 h at 4 °C with gentle rocking. Beads were collected by quick-spin centrifugation, and the supernatants removed. Affinity-purified antibodies (1 μg) were added to the supernatants and incubated for 2 h at 4 °C with gentle rocking. Forty microliters of 50 % protein A-agarose slurry was added and incubation continued for 2 h at 4 °C. Beads were washed three times for 15 min with 1 ml of NETT buffer at 4 °C by gentle rocking and were then equilibrated in 50 μl of kinase buffer (50 mM Tris–HCl pH 7.5, 10 mM MgCl_2_, 20 mM EGTA, 1 mM DTT, and 1 mM β-glycerophosphate) on ice. Following removal of the supernatant, a mixture containing 7 μl of kinase buffer and 1 μl each of 2.5 μg/μl of histone H1 (Sigma) or GST-E2F1 (Sabelli et al. 2005), 4 mM ATP, and 10 mCi/ml γ[^32^P]ATP (370 MBq/ml) (Amersham) was added to the beads. Reactions were carried out for 30 min at room temperature and stopped with 10 μl of 5× SDS-PAGE sample loading buffer. Reaction products were separated by 12.5 % SDS-PAGE. Gels were stained with Coomassie Blue R250, dried, and exposed to a Phosphorscreen, which was scanned with a Storm 860 Phosphorimager. Quantitation was performed with ImageQuant. Control assays using purified IgG from pre-immune sera were simultaneously performed for each developmental stage, and the resulting background histone H1 phosphorylation was subtracted from the phosphorylation obtained with cyclin-specific, affinity-purified IgG.

### In vitro assay of cyclin stability

Inhibition of proteasome-dependent proteolytic activity was performed with carboben-zoxyl-leucinyl-leucinyl-leucinal (MG-132, Sigma). Reactions (final volume 30 μl) contained endosperm extracts (50 μg of total soluble protein) pre-incubated with 100 μM MG-132 dissolved in dimethylsulfoxide or dimethylsulfoxide alone (control) on ice for 15 min, to which 1–3 μl of [^35^S]-labeled, in vitro-translated protein, 2 μg of poly-histidine-tagged human ubiquitin (Sigma), 0.1 units of phosphocreatine kinase, and ATP and phosphocreatine at final concentrations of 3 mM and 10 mM, respectively, were added. Reactions for assessment of proteasomal-mediated protein degradation in 7-DAP extracts contained Complete EDTA-free Protease Inhibitor Cocktail (Roche). Reactions were incubated at 30 °C and terminated at various times by adding SDS-PAGE sample loading buffer. Reaction products were resolved by SDS-PAGE; gels were fixed, dried and exposed to X-ray film with intensifying screens at −80 °C.

## Results

### Identification and characterization of cyclins and CDKs expressed in maize endosperm

We searched Pioneer Hi-Bred’s and public databases to identify D-, A- and B-type cyclins and A- and B-type CDKs potentially expressed during maize endosperm development. The identities and features of cyclins and CDKs thus identified are presented in Table 1. Among the identified cyclins are representatives of the A-, B- and D-types that were previously reported. CYCA1;1 and CYCA1;2 were originally named cycIIZm (Renaudin et al. 1994) and cycZm2w (Hsieh et al. 1998), respectively, and are 82 % identical at the amino acid sequence level. CYCA1;1 and an additional maize A1-type cyclin that we previously designated as CYCA1;3 (Coelho et al. 2005; Sabelli et al. 2005) have nearly identical sequences, are probably allelic and thus both were named CYCA1;1. Maize CYCB1;3 was previously proposed to be a mitotic cyclin (Sun et al. 1997, 1999). CYCD2;1, CYCD5;1 and the closely related (75 % identical at the amino acid level) CYCD5;2 were previously characterized with respect to their expression and CDK-associated activity during seed germination (Gutiérrez et al. 2005; Quiroz-Figueroa and Vázquez-Ramos 2006; Lara-Núñez et al. 2008). Computational predictions of subcellular localization indicated with higher reliability that CYCB1;3 is localized to cytosol, chloroplast and nucleus, CYCD2;1 to cytosol and nucleus, and CYCD5;1 and CYCD5;2 collectively to endoplasmic reticulum, chloroplast and, to a lesser extent, nucleus and mitochondrion (Table 1).

**Table 1:** Identity and features of cyclins and CDKs investigated in this work

Gene	Gene ID^c^	References^d^	Function^e^	Predicted subcellular localization^f^
*CYCA1;1* ^a^	GRMZM2G017081	Renaudin et al. (1994)	G2, M	–
*CYCA1;2* ^a^	GRMZM2G007113	Hsieh et al. (1998)	S, G2	–
*CYCB1;3*	GRMZM2G005619	Sun et al. (1999)	G2, M	C (cytosol, chloroplast); N
*CYCD2;1*	GRMZM2G075117	Gutiérrez et al. (2005)	G1, S	C (cytosol); N
*CYCD5;1* ^b^	GRMZM2G006721	Quiroz-Figueroa and Vázquez-Ramos (2006)	Constitutive	C (chloroplast, mitochondrion); N
*CYCD5;2* ^b^	GRMZM2G007130	Quiroz-Figueroa and Vázquez-Ramos (2006)	G1, S	C (endoplasmic reticulum, cytosol)
*CDKA;1*	GRMZM2G008327	Colasanti et al. (1991)	Constitutive	–
*CDKA;3*	GRMZM2G174596	This work	–	–
*CDKB1;1*	GRMZM2G495626	This work	–	–

*C* cytoplasm, *N* nucleus

^a^CYCA1;1 and CYCA1;2 proteins from maize tissue samples are collectively referred to as CYCA1 in this work

^b^CYCD5;1 and CYCD5;2 proteins and RNAs from maize tissue samples are collectively referred to as CYCD5 in this work

^c^Gene ID assigned by the Maize Genome Sequencing Project

^d^Originally reported sequence and expression analyses

^e^Cell cycles phases at which a function was assigned in previous studies

^f^Most reliable computational predictions of subcellular localization of proteins that were characterized via immunohistochemistry in this work

Two nearly identical maize A-type CDKs were previously described, CDKA;1 and CDKA;2, which were originally named cdc2ZmA and cdc2ZmB, respectively (Colasanti et al. 1991). However, we also identified previously uncharacterized A- and B1-type CDKs. An additional PSTAIRE-containing CDK (GenBank accession number AX040966), which we designated CDKA;3, is 92 % identical to rice CDKA;2 (formerly cdc2Os2) (Hashimoto et al. 1992), and is 81 % identical to maize CDKA;1 and CDKA;2. A B-type CDK (AX040970), which we named CDKB1;1, contains a PPTALRE motif and is 81 % identical to rice CDKB1;1 (Guo et al. 2007). Both maize CDKA;3 and CDKB1;1 sequences contain conserved amino acid residues that are typically involved with ATP binding and regulation of kinase activity across different kingdoms.

### Differential expression patterns of cyclins and CDKs during maize endosperm development

**Fig. 1:**
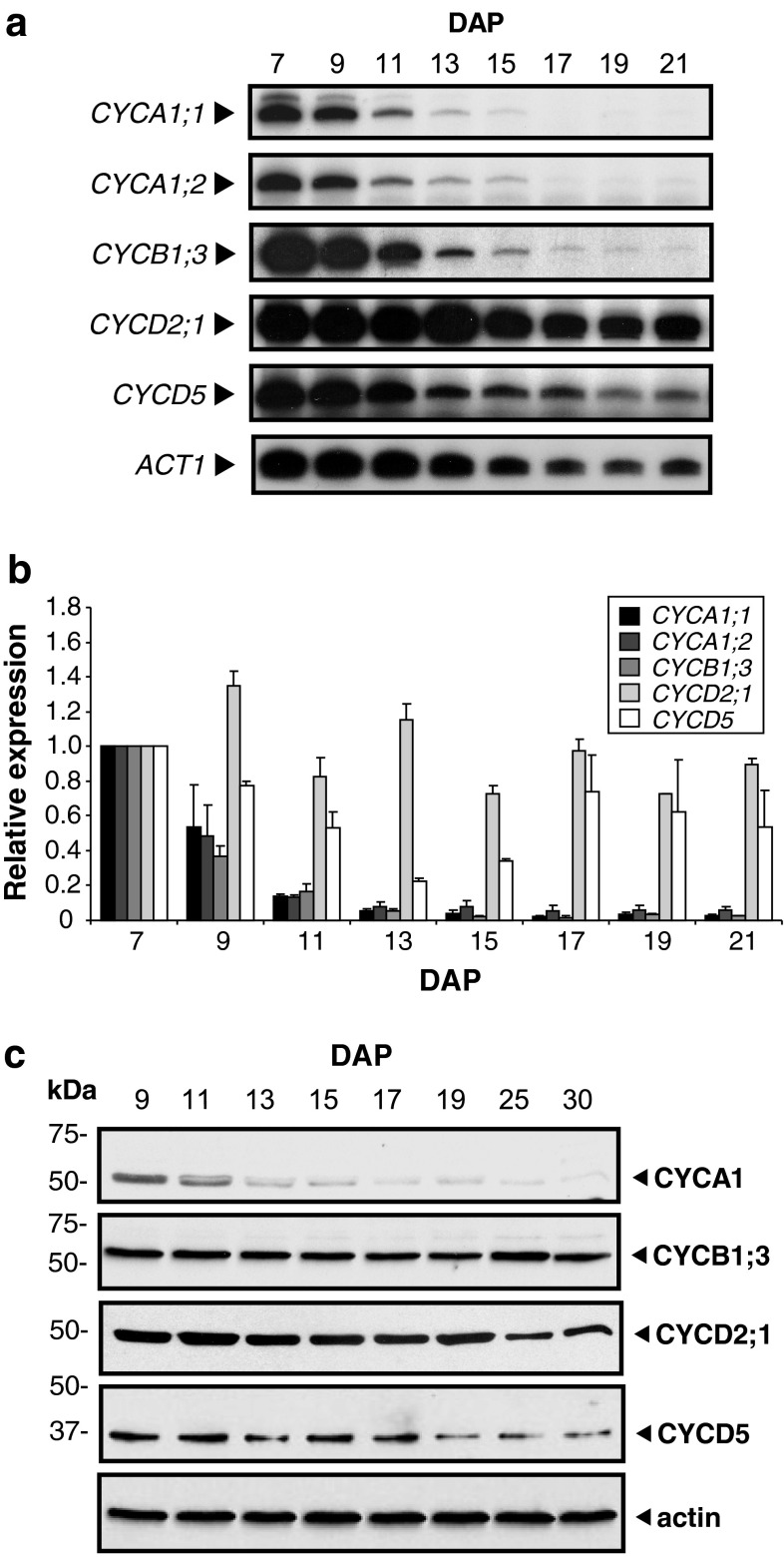
Cyclin expression levels during maize endosperm development. **a** Cyclin and actin RNA levels during endosperm development. RT-PCR was performed on total RNA isolated from endosperm at different developmental stages (indicated by DAP) with cyclin- or *ACT1*-specific primers. Reaction products were separated by electrophoresis and exposed to a Phosphorscreen. **b** Cyclin RNA levels relative to actin RNA in developing endosperms. Signal from amplicons from two independent experiments was quantified, and expression was normalized relative to 7 DAP, which is considered one expression unit. *Error bars* show standard error of the means. **c** Immunoblot analysis of cyclins during maize endosperm development. Total soluble protein extracted from endosperm at different developmental stages (indicated by DAP) was separated by SDS-PAGE and immunoblotted with affinity-purified antibodies as indicated on right. An immunoblot with actin antibody is shown as the loading control

To determine the expression patterns of cyclin transcripts, RT-PCR analyses (Fig. 1) were performed with RNA extracted from endosperm at 7 through 21 DAP. Flow cytometric analyses revealed that during this developmental period endosperms were initially at the mitotic cell cycle stage (7 DAP), progressed through a mitotic cell cycle-to-endoreduplication transition stage (9–13 DAP), and finally reached the endoreduplication stage (after 13 DAP) (Online resource 2). Cyclin and CDK transcript levels were measured relative to those of actin (*ACT1*) to minimize the RNA diluting effect caused by the massive expression of storage protein genes after 10 DAP. *CYCA1;1* and *CYCA1;2* transcripts were highly expressed at 7 DAP and were then rapidly down-regulated (Fig. 1a, b). Likewise, *CYCB1*;*3* transcript levels were highest at the mitotic stage of endosperm development and decreased dramatically thereafter, consistent with previous observations (Sun et al. 1999). Because during the course of this work we were unaware of *CYCD5;2* and its sequence similarity to *CYCD5;1*, their individual transcript levels were not distinguished and are represented as *CYCD5*. Levels of *CYCD5* transcripts were highest during early and late developmental stages. *CYCD2;1* transcript levels peaked at 9 DAP and became only marginally reduced throughout the remainder of endosperm development.

To investigate the potential role of the different types of cyclins during maize endosperm development, we analyzed their expression and localization at the cellular level, as well as their associated CDK activities. Polyclonal antibodies against unique N- or C-terminal regions of selected cyclins were affinity purified and their specificity confirmed by immunoblotting with recombinant cyclins and endosperm extracts (Online resource 3). Antibodies raised against CYCD5;1 would probably recognize CYCD5;2 because of their high sequence identity. Consequently, their combined protein expression in maize tissue samples is represented as CYCD5. Similarly, antibodies raised against CYCA1;1 could also recognize CYCA1;2. Because CYCA1;1 and CYCA1;2 transcripts are similarly expressed during endosperm development (Fig. 1a, b), their combined protein expression in maize tissue samples is represented as CYCA1. To investigate the temporal expression patterns of CYCA1, CYCB1;3, CYCD2;1 and CYCD5 proteins during endosperm development, we performed immunoblot analysis with total soluble protein extract from 9- to 30-DAP endosperms (Fig. 1c). With the exception of CYCB1;3, all the cyclin protein accumulation patterns resembled those of their transcripts. CYCA1 protein levels declined rapidly after 9 DAP, as the endosperm developed. In contrast, the abundance of CYCD5 and CYCD2;1 proteins declined only slightly during endosperm development. Surprisingly, the CYCB1;3 protein level remained relatively constant, although its transcript levels became dramatically reduced (Fig. 1a, b; Sun et al. 1999).

**Fig. 2:**
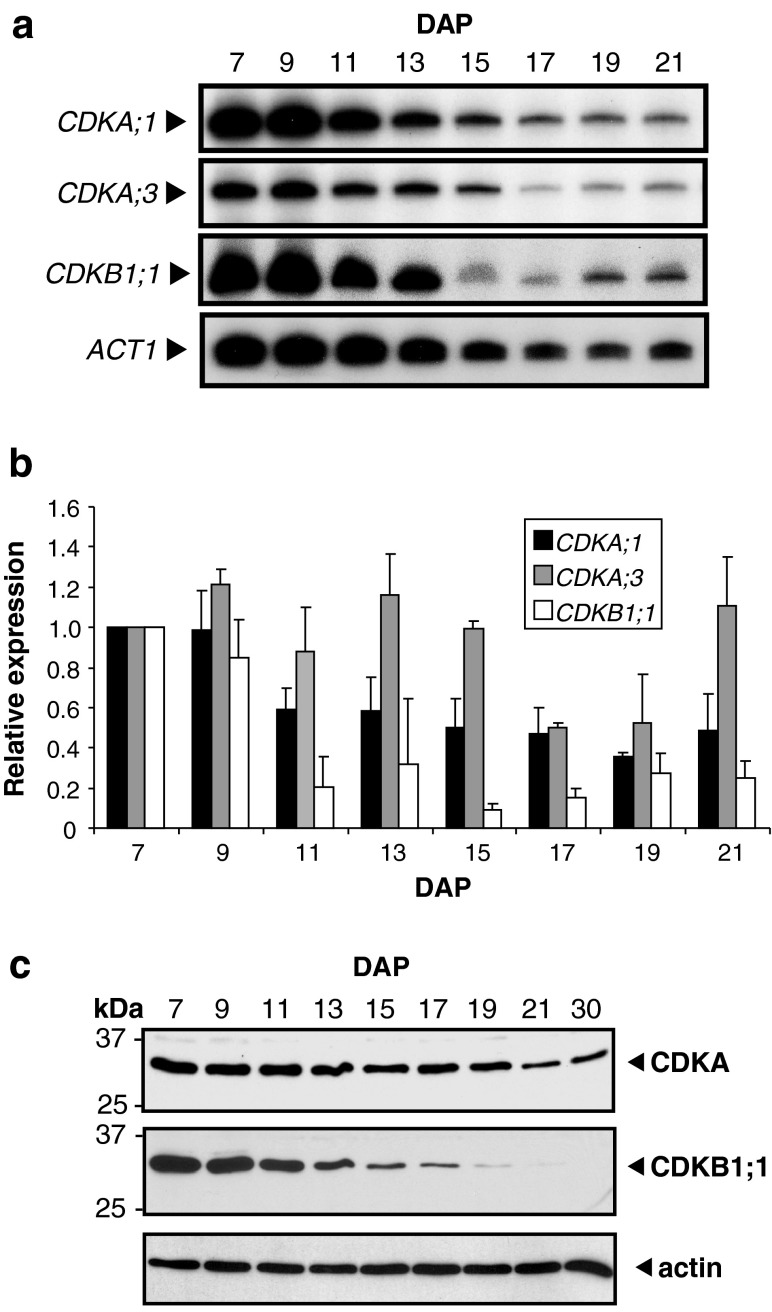
CDK expression levels during maize endosperm development. **a** CDK and actin RNA levels during endosperm development. RT-PCR was performed on total RNA isolated from endosperm at different developmental stages (indicated by DAP) with CDK- or *ACT1*-specific primers. Reaction products were separated by electrophoresis and exposed to a Phosphorscreen. **b** CDK RNA levels relative to actin RNA in developing endosperms. Signal from amplicons from two independent experiments was quantified and expression was normalized relative to 7 DAP, which is considered one expression unit. *Error bars* show standard error of the means. **c** Immunoblot analysis of CDKs present during maize endosperm development. Total soluble protein extracted from endosperm at different developmental stages (indicated by DAP) was separated by SDS-PAGE and immunoblotted with affinity-purified antibodies as indicated on the right. An immunoblot with actin antibody is shown as the loading control

CDK transcript levels during the same period of endosperm development were likewise analyzed by RT-PCR analysis (Fig. 2a, b). *CDKA;1* and *CDKA;3* transcript levels were high at 7–9 DAP, but while CDKA;3 transcript levels were similarly expressed at most stages of endosperm development, *CDKA;1* transcript levels appeared to decline slightly more rapidly after 9 DAP. In contrast, *CDKB1;1* transcript levels were highest in 7–9-DAP endosperm, and declined markedly at later stages of development.

Polyclonal antibodies were also raised against full-length CDKA;1 and CDKB1;1 proteins and affinity purified. These antibodies recognized specifically A-type CDKs or CDKB1;1 (Online resource 4). Immunoblot analyses of endosperm extracts showed that a single band of approximately 34 kDa was recognized by CDKA;1 antibodies in endosperm extracts (Fig. 2c). Because both CDKA;1 and CDKA;3 were recognized by CDKA;1 antibodies (Online resource 4), and CDKA;1 and CDKA;3 transcript expression patterns were relatively similar during endosperm development (Fig. 2a, b), the single 34-kDa band likely represents both CDKA;1 and CDKA;3 polypeptides. A-type CDK levels were highest at 7 DAP, declined only slightly during endosperm development, but they were still clearly detectable as late as 30 DAP. CDKB1;1 antibodies also recognized a polypeptide of approximately 34 kDa in endosperm extracts (Fig. 2c). CDKB1;1 protein levels were highest at 7 DAP, declined rather rapidly after 9–11 DAP, and were hardly detectable after 17 DAP. Thus, the expression pattern of CDKB1;1 protein matched closely that of its transcript.

### Different cyclins display unique tissue and subcellular localization patterns during endosperm development

**Fig. 3:**
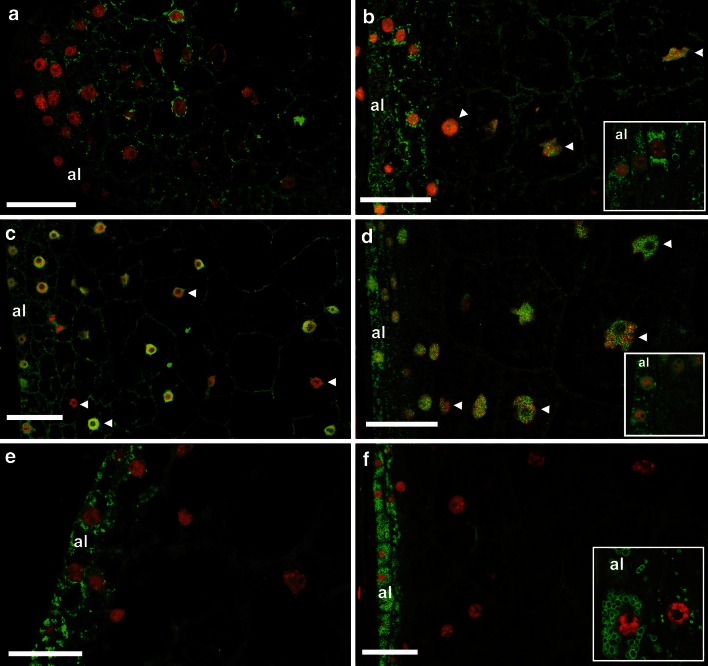
Localization of cyclins in mitotic and endoreduplicating endosperm cells. Longitudinal sections of kernels at mitotic (**a**, **c**, **e**) and endoreduplication (**b**, **d**, **f**) stages of endosperm development were immunostained with CYCB1;3 (**a**, **b**), CYCD2;1 (**c**, **d**), and CYCD5 (**e**, **f**) antibodies (shown in *green*). DNA was stained with To-Pro 3-iodide^®^ (shown in *red*). Developmental stages were 5 DAP (**a**), 7 DAP (**c**, **e**), 15 DAP (**b**), and 13 DAP (**d**, **f**). *Arrowheads* in **b**, **c** and **d** indicate nuclei with different intensities of cyclin labeling. Higher magnification *insets* illustrate subcellular localization patterns discussed in the text. *al* aleurone. *Bars* 50 μm

To address whether the expression of different cyclin proteins occurs in mitotic or endoreduplicating cells, we determined the spatial localization of CYCB1;3, CYCD2;1 and CYCD5 proteins in developing endosperm. Because CYCA1;1 antibodies cross-reacted with unknown polypeptides in immunoblots of endosperm protein extracts (Online resource 3), determining the localization of CYCA1;1 was not pursued. Indirect immunofluorescence assays of 5- or 7-DAP (mitotic stage) and 13- or 15-DAP (endoreduplication stage) kernel sections revealed distinct patterns of cyclin localization in interphase cells with respect to the endosperm region and stage of development, as well as the cellular compartment (Fig. 3). During both the mitotic and endoreduplication stages, CYCB1;3 (Fig. 3a, b) and CYCD2;1 proteins (Fig. 3c, d) were detected throughout the endosperm, while CYCD5 was detected only in peripheral cell layers (aleurone and subaleurone) (Fig. 3e, f). Immunohistochemical analyses of cyclin expression were generally consistent with computational predictions of subcellular localization and immunoblot analysis of cell fractions (Online resource 5).

At the mitotic stage (Fig. 3a), CYCB1;3 was detected mostly in perinuclear regions and along cytoplasmic strands throughout the endosperm. In contrast, at the endoreduplication stage (Fig. 3b) it was localized in both the cytoplasm and nuclei, most conspicuously in the aleurone and subaleurone layers, where it appeared as cytoplasmic aggregates and in ring-like structures (inset). In the enlarged central endosperm cells, fewer of these aggregates were found, and CYCB1;3 was distributed diffusely between starch granules. Also at the endoreduplication stage, variable levels of CYCB1;3 were detected in different nuclei (indicated by arrowheads).

In contrast to CYCB1;3, which was primarily cytoplasmic, CYCD2;1 displayed both cytoplasmic and nuclear localization (Fig. 3c) at the mitotic stage of development. Nuclei displayed different degrees of CYCD2;1 staining (indicated by arrowheads). There appeared to be no major differences in CYCD2;1 localization between the peripheral and the central endosperm at this stage. In contrast to mitotic endosperm (Fig. 3c), in endoreduplicating endosperm (Fig. 3d) CYCD2;1 was found in cytoplasmic aggregates, primarily in the three outermost cell layers (inset) with a gradient of labeling that decreased centripetally, and distributed diffusely between starch granules in enlarged central cells. Also at the endoreduplication stage CYCD2;1 showed nuclear localization, which was variable in central endosperm cells (indicated by arrowheads).

Unlike CYCB1;3 and CYCD2;1, which were found in both peripheral and internal starchy endosperm cells, CYCD5 was restricted to peripheral cells both in mitotic and endoreduplicating endosperm. At the mitotic (Fig. 3e) and the endoreduplication (Fig. 3f) stages, CYCD5 was limited to aleurone and subaleurone cells, where it was found in the cytoplasm but never in nuclei. CYCD5 occurred along cytoplasmic strands and in ring-like structures around nuclei, which became more evident in endoreduplicating endosperm (inset).

### CDKB1;1 expression is increased in endosperm upon stimulation of the G1/S transition

**Fig. 4:**
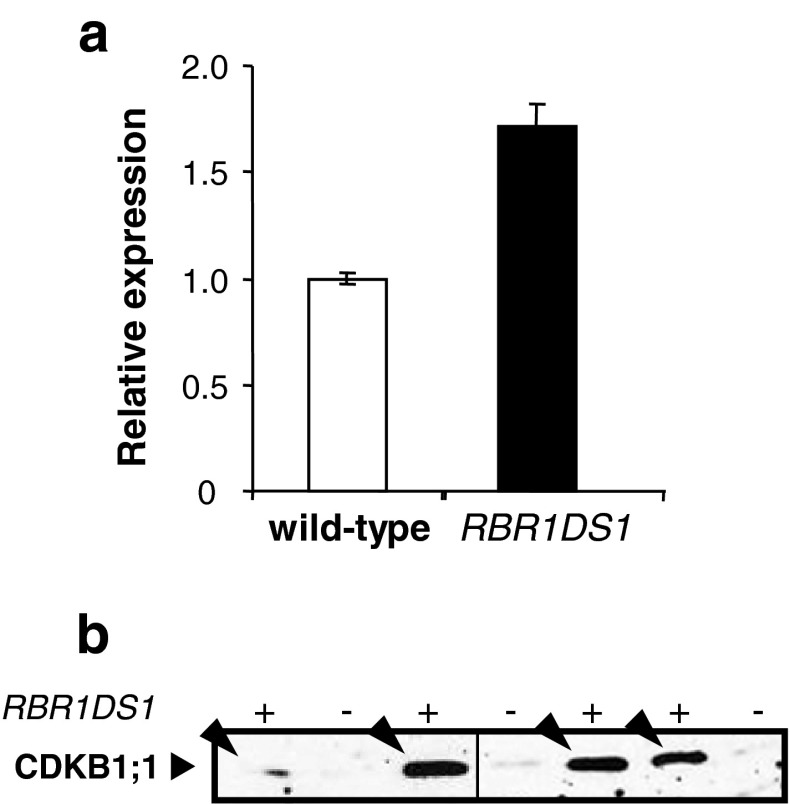
Expression of CDKB1;1 in wild-type (−) and *RBR1DS1* (+) endosperms. **a** Quantitative RT-PCR analysis of *CDKB1;1* transcript abundance was performed on 16-DAP endosperms. *Error bars* show standard errors of the means. **b** Immunoblot analysis was performed on individual, segregating *RBR1DS1* and wild-type 16-DAP endosperms. Up-regulated CDKB1;1 in *RBR1DS1* endosperms is indicated by arrowheads. Equal sample loading was verified via actin immunoblotting and reported by Sabelli et al. (2013)

In the endosperm of the maize *RBR1DS1* transgenic line, the expression of RBR1 is reduced via RNAi, with consequent enhancement of downstream E2F/DP-dependent gene expression and increase of both cell number and nuclear ploidy (Sabelli et al. 2013). In Arabidopsis, overexpression of an E2F/DP transcription factor stimulates both cell division and endoreduplication (De Veylder et al. 2002), CDKB1;1 expression, which is E2F/DP-dependent, and influences the shift between cell division and endoreduplication cycles (Boudolf et al. 2004). Whether CDKB1;1 is a transcriptional target of E2F/DP (and thus, repressed by RBR1) in maize is not known. Consequently, we investigated whether CDKB1;1 expression is modified, in association with stimulated E2F/DP-dependent gene expression and cell cycle progression, in 16-DAP *RBR1DS1* endosperm. *CDKB1;1* transcript levels were 1.7-fold higher in *RBR1DS1* endosperm compared to wild-type endosperm (Fig. 4a). Similarly, CDKB1;1 protein, which is expressed at low levels at this stage of development in wild-type endosperm, was clearly up-regulated in *RBR1DS1* endosperm (Fig. 4b). These results indicate that stimulation of the G1/S transition is associated with increased CDKB1;1 expression in maize endosperm cells, and provide genetic evidence that CDKB1;1 is an E2F/DP/RBR1 target.

### Cyclin binding and A-type CDK activation in Drosophila S2 cells

**Fig. 5:**
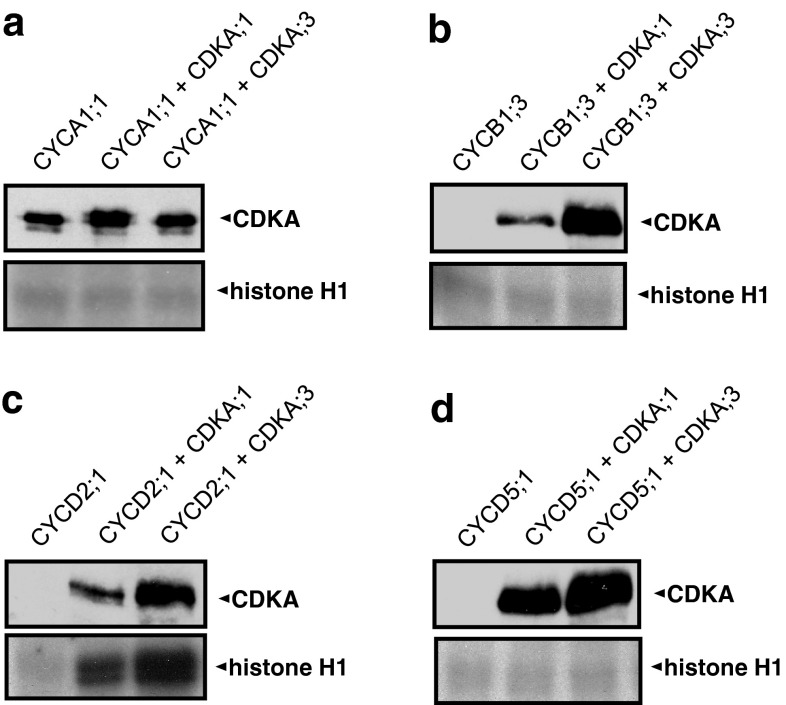
Interaction of CDKA;1 and CDKA;3 with cyclins and assembly of active kinase complexes. Maize CDKAs and cyclins were co-expressed in Drosophila S2 cells and complexes were immunoprecipitated from extracts with the corresponding cyclin antibodies. Immunoprecipitates were analyzed by immunoblotting with a PSTAIR-motif antibody (*upper panel* in each figure) and histone H1 kinase assays (*lower panel*). **a** In vitro-synthesized CYCA1;1 added to extracts of S2 cells expressing CDKA;1 or CDKA;3. Co-expression in S2 cells of CDKA;1 or CDKA;3 with CYCB1;3 (**b**), CYCD2;1 (**c**) and CYCD5;1 (**d**)

To investigate the composition of the complexes formed by the different CDK and cyclin proteins studied in this work, we tested the ability of CDKA;1, CDKA;3 and CDKB1;1 to physically interact with different maize cyclins in Drosophila S2 cells (Fig. 5). Because CYCA1;1 could not be expressed in S2 cells, in vitro-synthesized CYCA1;1 was added to extracts of S2 cells expressing the different CDKs. Cyclin immunoprecipitates were analyzed for the presence of CDKs by immunoblotting and assayed for kinase activity. In the case of co-expression assays with CDKA;1 and CDKA;3, the immunoprecipitates were immunoblotted with a mouse monoclonal PSTAIR antibody that detected A-type CDKs.

CDKB1;1 did not form active complexes with maize cyclins (data not shown), and because of the background signal from the cyclin antibodies used for immunoprecipitation, we could not determine whether CDKB1;1 bound any of the maize cyclins. A-type CDKs expressed in combination with cyclins in S2 cells could be immunoprecipitated successfully with the corresponding cyclin antibodies, demonstrating that they do not interfere with the ability of cyclins to interact with CDK partners. CYCA1;1 interacted with a ~34 kDa, PSTAIRE-containing polypeptide not only in extracts of cells expressing CDKA;1 or CDKA;3, but also in control extracts (Fig. 5a). These are likely the endogenous Drosophila PSTAIRE-containing CDK1 and/or CDK2. Therefore, we cannot unambiguously establish whether maize CDKA;1 or CDKA1;3 bound CYCA1;1 in these assays. CYCB1;3 (Fig. 5b) and CYCD5;1 (Fig. 5d) interacted with CDKA;1 and CDKA;3, although no active kinase complexes were formed. However, CYCD2;1 interacted with CDKA;1 or CDKA;3, producing active kinase complexes that phosphorylated histone H1 (Fig. 5c). The level of kinase activity observed appeared to be proportional to the amount of CDKA/cyclin complex formed. The results indicate that CDKA;1 and CDKA;3 have similar abilities to interact with CYCB1;3, CYCD5;1 and CYCD2;1 and form catalytically active kinase complexes with CYCD2;1 in vivo.

### Kinase activities associated with different cyclins vary during endosperm development

**Fig. 6:**
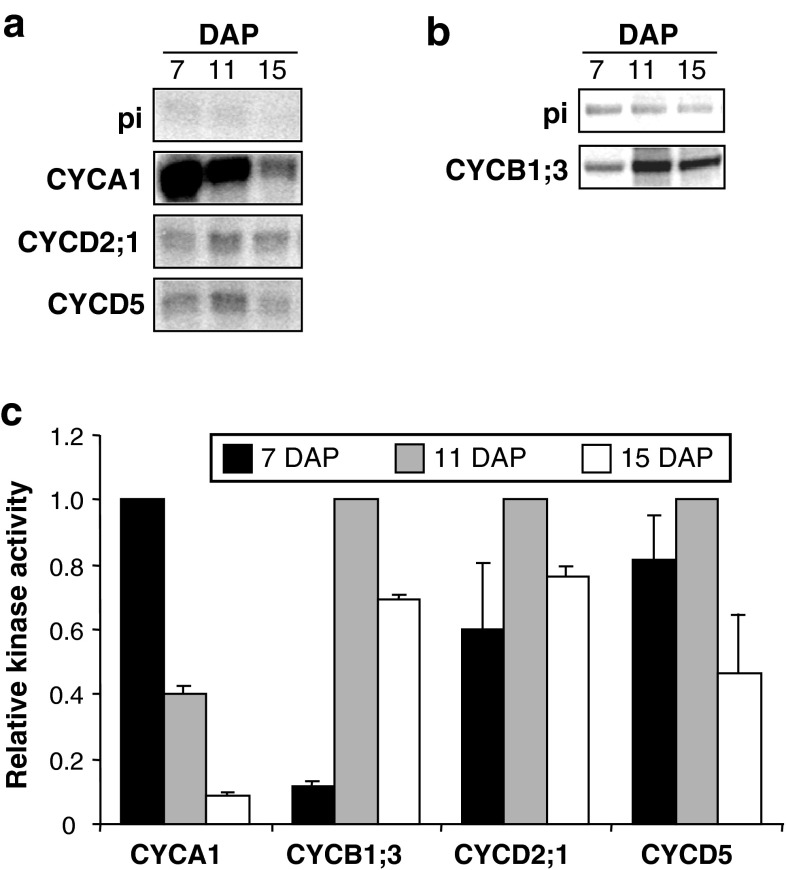
Kinase activity associated with CYCA1, CYCB1;3, CYCD5, and CYCD2;1 in developing maize endosperm. Cyclins were immunoprecipitated from endosperm extracts with specific antibodies, and the activity of the associated kinase was measured by phosphorylation of histone H1 (CYCA1, CYCD5, and CYCD2) or GST-E2F1 (CYCB1;3) substrates. **a** Autoradiographic detection of histone H1 phosphorylated by CYCA1-, CYCD5-, and CYCD2;1-associated kinases. Equal exposures of control assays that contained IgG from pre-immune serum instead of cyclin antibodies are shown (pi). **b** Autoradiographic detection of GST-E2F1 phosphorylated by CYCB1;3-associated kinase. Equal exposures of control assays are shown (pi). **c** Relative kinase activity at 7, 11, and 15 DAP. Values are averages from two independent experiments and were normalized to those from the developmental stages displaying the highest kinase activity for each cyclin, which are considered as one expression unit. *Error bars* show standard deviations of the means

Since D-, A- and B-type cyclins characteristically control progression through distinct phases of the cell cycle, we investigated whether the amount of kinase activity associated with CYCA1, CYCB1;3, CYCD2;1 and CYCD5 differed in endosperm at 7 DAP (mitotic stage), 11 DAP (mitotic to endoreduplication transition stage), and 15 DAP (endoreduplication stage). Kinase assays were performed on CYCA1, CYCD2;1 and CYCD5 immunoprecipitates using histone H1 as substrate (Fig. 6). Since higher levels of CYCB1;3-associated kinase activity were obtained with a maize E2F1 homolog fused to GST (Sabelli et al. 2005) as substrate, histone H1 was replaced by GST-E2F1 in CYCB1;3 kinase activity assays.

These experiments revealed that the amount of kinase activity recovered varied depending on the cyclin type and developmental stage (Fig. 6). CYCA1-associated activity was highest at 7 DAP, and it was reduced to about 40 and 7 % of this level at 11 and 15 DAP, respectively (Fig. 6a, c). In contrast, the kinase activity associated with CYCB1;3 (Fig. 6b, c), CYCD5 and CYCD2;1 (Fig. 6a, c) peaked at 11 DAP. Compared to the kinase activity present at 11 DAP, the amount of CYCB1;3-associated activity was much lower at 7 DAP (11 %) than at 15 DAP (69 %) (Fig. 6b, c). Also, compared to 11-DAP levels, the amount of CYCD5;1-associated kinase activity was higher at 7 DAP (81 %) than at 15 DAP (47 %), while that associated with CYCD2;1 was only slightly lower at 7 DAP (60 %) than at 15 DAP (76 %) (Fig. 6a, c). Compared to the kinase activity associated with CYCA1 and CYCB1;3, that associated with CYCD5 and CYCD2;1 appeared to be more constant throughout the developmental stages examined. These results indicate that the activity of CYCA1-containing kinase complexes is highest at developmental stages where most cells are dividing rather than endoreduplicating. On the other hand, the activity of CYCB1;3-, CYCD5- and CYCD2;1-containing complexes appeared to be highest at 11 DAP, corresponding approximately to the mitosis/endoreduplication transition stage, when the rates of cell division and nuclear ploidy increase are maximal.

### Proteasome-dependent degradation of cyclins is decreased in endoreduplicating endosperm cells

**Fig. 7:**
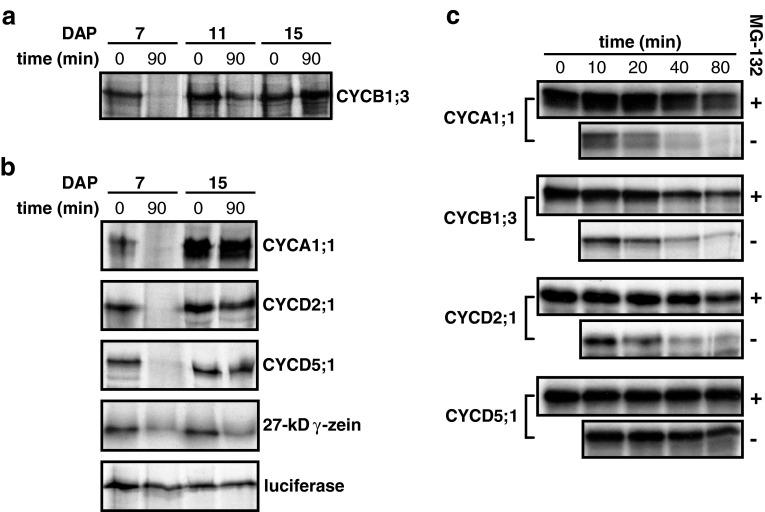
Cyclin stability in extracts from mitotic and endoreduplicating endosperm cells. Radio-labeled proteins synthesized in vitro were incubated with extracts from endosperm at different stages of development. Reaction products were separated by SDS-PAGE, and gels were exposed to X-ray films. Incubation times are indicated in minutes. **a** Stability of CYCB1;3 in 7-, 11-, and 15-DAP endosperm extracts. **b** Stability of CYCA1;1, CYCD5;1, CYCD2;1, 27-kDa γ-zein, and firefly luciferase in 7- and 15-DAP endosperm extracts. **c** Time-course analysis of cyclin degradation in 7-DAP endosperm extract and its inhibition by the proteasome inhibitor, MG-132; (+) and (−) symbols indicate presence or absence of MG-132, respectively

The relationship among CYCB1;3 transcript levels (Fig. 1a, b; Sun et al. 1999), CYCB1;3 protein levels (Fig. 1c), and CYCB1;3-associated kinase activity (Fig. 6), and the presence of CYCB1;3 in endoreduplicated cells (Fig. 3b) indicated that CYCB1;3 protein is relatively stable in endoreduplicating endosperm cells. As this was unexpected, we investigated the basis for CYCB1;3 stability by performing a degradation assay combining mitotic (7 DAP), mitotic-transitioning-to-endoreduplicating (11 DAP) or endoreduplicating (15 DAP) endosperm extracts with in vitro-translated, radio-labeled CYCB1;3 (Fig. 7a). Extract of mitotic, but not endoreduplicating endosperm, degraded CYCB1;3 during a 90-min incubation in vitro. Extract from 11-DAP endosperm, a stage at which both mitotic and endoreduplication cell cycles occur, only partially degraded CYCB1;3. This result suggested that the proteolytic activity that degraded CYCB1;3 at 7 DAP gradually disappeared with the onset of endoreduplication.

We next investigated whether other cyclins also showed differential stability in 7- and 15-DAP endosperm cell extracts (Fig. 7b). The results showed that CYCA1;1, CYCD5;1 and CYCD2;1, similarly to CYCB1;3, were all degraded by mitotic but not endoreduplicating extracts, suggesting that mitotic but not endoreduplicating endosperm cells possess the ability to degrade different types of cyclins. To investigate whether this proteolytic activity was limited to cyclins, similar assays were performed using the 27-kDa γ-zein, an endosperm storage protein, and firefly luciferase as substrates (Fig. 7b). The 27-kDa γ-zein was degraded to a similar extent in both 7- and 15-DAP endosperm extracts, whereas luciferase was not affected in either. This result suggested that cyclins are specifically resistant to the proteolytic activity that exists in 15-DAP endosperm extract, and that cyclin proteolysis in 7-DAP endosperm extracts was caused by a rather specific proteolytic activity.

To determine whether cyclin degradation in 7-DAP endosperm extracts was proteasome dependent, in vitro-translated cyclins were incubated with endosperm extract containing a protease inhibitor cocktail that minimized the contribution of general, non-proteasomal proteolytic activity. As shown in Fig. 7c, in control reactions lacking the proteasome-specific inhibitor, MG-132, CYCA1;1, CYCB1;3, and CYCD2;1 were progressively degraded by 7-DAP endosperm extracts (panels labeled with a minus symbol), although CYCD5;1 was relatively more stable. Addition of MG-132 considerably inhibited the proteolysis of all four types of cyclins in 7-DAP extract (panels labeled with a plus symbol). These results indicate that in endosperm cells undergoing mitotic cell cycles, the ubiquitin/26S proteasome pathway mediates cyclin degradation, but that proteasome-dependent cyclin degradation becomes significantly less pronounced as these cells switch to the endoreduplication cell cycle.

## Discussion

### Cyclin proteins show distinct spatiotemporal expression patterns in developing maize endosperm

This study shows that expression of different types of maize cyclins is specifically regulated, both temporally and spatially, during endosperm development. The temporal expression patterns of CYCA1, CYCD2;1, and CYCD5 RNA and protein are in accordance with the presumed function of these genes during the mitotic and endoreduplication cell cycles. Unexpectedly, however, CYCB1;3 protein was present at nearly constant levels throughout endosperm development. This is surprising in view of the observed sharp reduction of its transcript at the onset of endoreduplication (Fig. 1), which is in agreement with data from previous studies and its expected function during M-phase (Sun et al. 1997, 1999). Thus, unlike CYCA1, CYCD2;1 and CYCD5, which exhibited reasonably matching transcript and protein expression patterns, CYCB1;3 protein expression seemed to be, to a large extent, positively regulated at the translational and/or post-translational levels during the endoreduplication stage of development.

Immunohistochemical analyses revealed complex tissue and subcellular localization patterns for CYCB1;3 and CYCD2;1 in developing endosperm. At both the mitotic and endoreduplication stages of development, these proteins exhibited wide tissue distribution. The degree to which they were localized dynamically in the nuclear and cytoplasmic compartments differed among cells, which is indicative of cell cycle-regulated expression within a tissue containing cells that progress through the cell cycle phases asynchronously. At the endoreduplication stage, CYCB1;3 and CYCD2;1 exhibited substantial cytoplasmic localization in peripheral mitotic cells, while in inner endoreduplicated cells these proteins were more clearly localized to nuclei rather than cytoplasm (Fig. 3b, d). Thus, sustained CYCB1;3 protein expression late in development (Fig. 1c) is possibly due to its persistence not only in the cytoplasm of peripheral mitotic cells but also in the nuclei of inner endoreduplicated cells, consistent with immunoblot analysis of cell fractions (Online resource 5). Previous immunohistochemical analyses of maize roots revealed that a B1-type cyclin, CYCB1;2, was completely absent from non-dividing cells while another B1-type cyclin, CYCB1;1, and CYCB2;1 disappeared from the cytoplasm, but persisted at low levels in nuclei of non-dividing, expanding cells (Mews et al. 1997, 2000). Therefore, it appears that, similar to expanding root cells, various types of cyclins are mostly localized to the nucleus of expanding endosperm cells.

In contrast to CYCB1;3 and CYCD2;1, CYCD5 was found in association with ring-like structures localized exclusively in the cytoplasm of aleurone and subaleurone cell layers at both the mitotic (Fig. 3e) and endoreduplication (Fig. 3f) stages of endosperm development. Thus, CYCD5 may be localized to endoplasmic reticulum, plastids or mitochondria in aleurone and subaleurone cells at these stages of development (Kyle and Styles 1977) and function in cytoplasmic processes associated with the mitotic cell cycle in these peripheral cells. Unlike maize, Arabidopsis possesses a single, nucleus-localized D5-type cyclin (Boruc et al. 2010) that is expressed in both dividing and endoreduplicating cells (Sterken et al. 2012). Thus, members of the D5-subtype of cyclins appear to play distinct roles in maize, potentially as a result of the higher complexity of the D-type cyclins in this species, which possesses particularly four members of the D5-subtype (Hu et al. 2010).

### Evidence for redundant and specialized roles of A-type CDKs and CDKB1;1 during endosperm development

A- and B1-type CDKs displayed different patterns of expression during endosperm development. While A-type CDK protein levels were largely constant at the mitotic and the endoreduplication stages, CDKB1;1 protein levels were substantially reduced during the endoreduplication stage (Fig. 2c). These expression patterns are consistent with a positive role of A-type CDKs in both the mitotic and endoreduplication cell cycles (Hemerly et al. 1995; Leiva-Neto et al. 2004), and with positive and negative roles of B1-type CDKs in the mitotic and endoreduplication cell cycles, respectively (Porceddu et al. 2001; Boudolf et al. 2004, 2009). Despite the specialized functions of A- and B1-type CDKs at different cell cycle phases, members of these CDK types were recently reported as partially redundant in Arabidopsis, in which B1-type CDKs have the ability to drive complete mitotic cycles in the absence of the single A-type CDK (Nowack et al. 2012). However, unlike Arabidopsis, maize possesses at least three A-type CDKs [the probable paralogs and nearly identical CDKA;1 and CDKA;2 (Colasanti et al. 1991) and CDKA;3], prompting the question as to whether they share redundant roles. Collectively, the results reported here and elsewhere (Leiva-Neto et al. 2004; Sabelli et al. 2013) suggest the existence of some degree of redundancy among A-type CDKs and a specialized role for CDKB1;1 in cell division in developing maize endosperm.

Possible redundancy between CDKA;1 and CDKA:3 is apparent from their relatively similar transcript expression profiles during endosperm development (Fig. 2) and at the global level (as revealed by transcriptomics analyses; not shown), and from their similar cyclin-binding activities in Drosophila S2 cells (Fig. 5). Severe reduction of RBR1 expression in endosperm results in increased p13^*suc1*^-associated kinase activity, cell number and nuclear ploidy (Sabelli et al. 2013). In contrast, overexpression of a dominant-negative form of CDKA;1 in endosperm reduces, but not abolishes, p13^*suc1*^-associated kinase activity and endoreduplication (Leiva-Neto et al. 2004). Importantly, elevated endoreduplication and p13^*suc1*^-associated kinase activity levels are also observed upon the concomitant down-regulation of RBR1 and CDKA;1 (Sabelli et al. 2013). Thus, additional CDKs seem to function redundantly (or in concert), at least partly, with CDKA;1 to suppress the cell cycle inhibitory function of RBR1 (Sabelli et al. 2013). The results presented here identify CDKA;3 as such a candidate CDK and underscore potentially important differences in cell cycle control resulting from higher A-type CDK complexity in species like maize, in comparison to species containing a single A-type CDK, such as *Arabidopsis thaliana*.

A positive role for maize CDKB1;1 in the mitotic cell cycle is likewise supported by the effects caused by reduced RBR1 expression in *RBR1DS1* endosperm, in which CDKB1;1 expression was increased (Fig. 4). Increased cell number and nuclear ploidy in *RBR1DS1* endosperm are consistent with the stimulation of the G1/S transition in mutually exclusive, dividing or endoreduplicating cells, across different endosperm regions and developmental stages. In Arabidopsis, several observations link the RBR/E2F/DP pathway, the regulation of CDKB1;1 expression, and the opposite effects of CDKB1;1 activity in the mitotic and endoreduplication cell cycles. Cells of plants overexpressing the E2Fa/DPa transcription factor can exhibit either ectopic division or enhanced endoreduplication phenotypes (De Veylder et al. 2002). *CDKB1;1* expression is E2F/DP dependent and CDKB1;1 activity levels determine whether cells undergo endoreduplication or, alternatively, divide mitotically depending on the presence of positive regulatory factors, presumably cyclins (Boudolf et al. 2004). Accordingly, overexpression of CDKB1;1, combined with that of its interacting partner CYCA2;3, triggers cell divisions and suppresses endoreduplication (Boudolf et al. 2009), and B1-type CDKs can drive progression through cell division, but not endoreduplication, upon depletion of CDKA;1 (Nowack et al. 2012). Therefore, maize CDKB1;1 could similarly stimulate cell proliferation and suppress endoreduplication in mitotically competent endosperm cells, probably depending upon its association with yet unidentified cyclins expressed in these cells.

### Composition, activity and potential function of CDK/cyclin complexes during maize endosperm development

Several lines of evidence indicate that CYCA1, CYCD5 and CYCD2;1 associate with CDKA;1 and/or CDKA;3 in developing endosperm. In contrast, the identity of the CYCB1;3-interacting CDK(s) is unknown. Inhibition of its associated kinase activity by CKIs indicates that CYCA1 forms catalytically active complexes with A-type CDKs in maize endosperm, since CYCA1-associated CDK activity is strongly inhibited by maize KRP;1 and KRP;2 (Coelho et al. 2005), which belong to the ICK/KRP-type CKIs that typically bind to and inhibit complexes containing A-type CDKs (reviewed by Wang et al. 2008). The residual CYCA1-associated activity in maize endosperm that is insensitive to inhibition by KRP;1 and KRP;2 could contain B-type CDKs, consistent with the interaction of Arabidopsis CYCA1;1 with A- and B-type CDKs in planta (Boruc et al. 2010). The association of A-type CDKs with various D-type cyclins in vivo was observed in several plant species. Particularly in maize, CYCD2;1 (Gutiérrez et al. 2005) and CYCD5 (Lara-Núñez et al. 2008) were co-immunoprecipitated with a PSTAIRE-containing CDK from germinating embryonic axes. In addition, CYCD2;1 and CYCD5;1 interacted with co-expressed CDKA;1 or CDKA;3 in Drosophila S2 cells (Fig. 5).

CYCA1 expression and its associated kinase activity were greatest during the mitotic stage of endosperm development (Fig. 6), suggesting a negative relationship with endoreduplication. This implies a role for CYCA1 in the G2/M transition of mitotic endosperm cells, which is consistent with the typical expression patterns of A1-type cyclins during the plant cell cycle. The expression of plant A-type cyclins occurs sequentially from late G1/early S-phase until mid M-phase (Reichheld et al. 1996) and appears to be positively associated with the mitotic cell cycle. Notably, Arabidopsis CYCA2;3 (Imai et al. 2006; Boudolf et al. 2009) and tobacco CYCA3;2 (Yu et al. 2003) suppress endoreduplication. Collectively, these observations suggest that down-regulation of CYCA1/CDK complexes, caused in part by reduced cyclin availability (Fig. 1) and by inhibition mediated by ICK/KRP-type CKIs (Coelho et al. 2005), could be involved in regulating the transition from a mitotic to an endoreduplication cell cycle in maize endosperm.

In comparison to CYCA1-associated kinase activity, that associated with CYCB1;3, CYCD5 and CYCD2;1 did not decline with the onset of endoreduplication. Rather, they peaked at 11 DAP, a developmental stage at which both the mitotic and endoreduplication cell cycles are highly active in the endosperm (Kowles and Phillips 1985; Lur and Setter 1993). Consistent with the roles of D-type cyclins in mediating the G1/S transition (reviewed by Inzé and De Veylder 2006), CYCD2;1- and CYCD5-associated activities were highest prior to cell division in germinating embryo axis cells (Gutiérrez et al. 2005; Lara-Núñez et al. 2008). In endosperm cells, CYCD2;1- and CYCD5-containing complexes could function similarly at the G1/S-phase transition. However, in contrast to Arabidopsis CYCD5;1, which is expressed in dividing and endoreduplicating cells and stimulates both cell cycles (Sterken et al. 2012), a role for CYCD5-containing CDK complexes in endosperm appears to be limited to mitotic cell cycles, since CYCD5 was found to localize mostly to peripheral cells (Fig. 3e, f), which retain mitotic activity until late developmental stages (Kiesselbach 1949) and typically do not undergo endoreduplication (Vilhar et al. 2002). Because CYCD2;1, on the contrary, was found in mitotic as well as endoreduplicated endosperm cells (Fig. 3c, d), its corresponding complexes with A-type CDKs could function at stimulating both the mitotic and endoreduplication cell cycles.

Altogether, CYCB1;3 localization and CYCB1;3-associated kinase activity during development suggest that CYCB1;3/CDK complexes function in both mitotic and endoreduplication cell cycles. The persistence of CYCB1;3 in central endosperm cells also suggests that, unexpectedly, the destruction of this protein is not required for endoreduplication, which instead could be related to the selective degradation of B-type cyclin(s) other than CYCB1;3. The function of maize CYCB1;3 in endoreduplicating endosperm cells could be equivalent to that of Arabidopsis CYCB1;1 in endoreduplicating trichomes, in which this cyclin is stably expressed (Roodbarkelari et al. 2010). In addition, CYCB1;3/CDK complexes could, at least in principle, participate in other endosperm developmental processes such as programmed cell death, which follows endoreduplication (Sabelli and Larkins 2009a; Sabelli 2012) and seems to be coordinately regulated with this cell cycle via *RBR1* (Sabelli et al. 2013). This possibility is supported by the observation that reduced proteolysis of B-type cyclins is associated with aberrant mitosis and subsequent programmed cell death in mammalian cells (reviewed by Vakifahmetoglu et al. 2008).

### A link between reduced proteasome-mediated degradation of cyclins, endoreduplication and cell expansion in maize endosperm

Evidence was provided in this work for substantially reduced 26S proteasome activity toward various types of cyclins in endoreduplicating endosperm compared to mitotic endosperm. This could contribute, along with other mechanisms of gene expression regulation, to increased availability of specific cyclins in endoreduplicating cells, most significantly in cases when protein levels remain constant, although transcript levels decline sharply, such as for CYCB1;3. Post-mitotic stabilization of A- and B-type cyclins in maize is not unprecedented, since it was observed in the root elongation zone (Mews et al. 1997, 2000). However, the responsible mechanisms remained unknown.

Proteolysis of A- and B-type cyclin proteins by the APC/C has been frequently observed in differentiated and endoreduplicated plant cells (reviewed by Marrocco et al. 2010; De Veylder et al. 2011; Heyman and De Veylder 2012). In Arabidopsis, the APC/C mediates the proteolysis of CYCA3;1 and CYCB1;1 from tobacco in several types of post-mitotic, differentiating cells, revealing sustained APC/C activity in the absence of a mitotic cell cycle program, which could prevent re-entry into the mitotic cell cycle (Marrocco et al. 2009). In contrast, the APC/C targets Arabidopsis CYCB1;1 only in mitotically active trichomes, but not those undergoing endoreduplication, causing their stabilization in the latter (Roodbarkelari et al. 2010). These observations suggest the existence of important differences in the APC/C-dependent proteolysis of various cyclins in distinct cell cycles and cell types. Consistent with this view, the contribution of the APC/C to the onset and maintenance of endoreduplication in plants is complex and variable, as revealed by functional analyses of individual APC/C subunits and co-activators (reviewed by Marrocco et al. 2010; Heyman and De Veylder 2012).

Reduced 26S proteasome-mediated proteolysis of A-, B- and D-type cyclins could contribute to endoreduplication and the concomitant cell expansion in maize endosperm. In agreement with our results, cell expansion and post-mitotic stabilization of CYCA1;1, CYCB1;1 and CYCB2;1 (Mews et al. 1997, 2000) and endoreduplication (Ogawa et al. 2010) all occur in the elongation zone of the maize root, further suggesting a link between reduced proteolysis of certain cyclins, cell expansion and endoreduplication. In support of this view, partial reduction of 26S proteasome activity in Arabidopsis leads to cell enlargement and increased endoreduplication in aerial organs (Kurepa et al. 2009; Sonoda et al. 2009).

In conclusion, this work suggests the existence of a developmentally regulated mechanism that reduces the proteasome-mediated degradation of cyclins belonging to the major, functionally distinct, A-, B-, and D-types in endoreduplicating and expanding endosperm cells. The wide range of cyclin types seemingly affected by reduced proteasomal activity raises the question as to whether additional cyclins and cell cycle regulators are similarly controlled by this mechanism, potentially creating extensive cell cycle modifications that are associated with endosperm cell expansion and endoreduplication. Further investigation will be required to identify the molecular basis for the differential activity of the ubiquitin/26S proteasome pathway in mitotic and endoreduplicating cells and determine its importance for endosperm development.

## Electronic supplementary material

Below is the link to the electronic supplementary material.

Electronic supplementary material The online version of this article contains supplementary material, which is available to authorized users.

Supplementary material 1 (PDF 210 kb)

## Abbreviations

APC/C: Anaphase promoting complex/cyclosome
BSA: Bovine serum albumin
CDK: Cyclin-dependent kinase
CKI: Cyclin-dependent kinase inhibitor
DAP: Days after pollination
DAPI: 4,6-diamidino-2-phenylindole
DMSO: Dimethyl sulfoxide
GST: Glutathione-*S*-transferase
ICK/KRP: Inhibitor of Cdc2 kinase/kip-related protein
MG-132: Carboben-zoxyl-leucinyl-leucinyl-leucinal
PBS: Phosphate buffered saline
PCR: Polymerase chain reaction
PIPES: Piperazine-*N*,*N*′-*bis*(2-ethanesulfonic acid)
RBR: Retinoblastoma-related
RT: Reverse transcription
