# Supplementary material for: Cyclin-dependent kinase complexes in developing maize endosperm: evidence for differential expression and functional specialization
Source: Planta. 2013 Nov 16;239(2):493–509. doi: 10.1007/s00425-013-1990-1 (PMC3902077; doi:10.1007/s00425-013-1990-1)
Supplement: Supplementary file 1 — Supplementary material 1 (PDF 210 kb) [file 425_2013_1990_MOESM1_ESM.pdf]

Cyclin-dependent kinase complexes in developing maize endosperm: evidence for differential expression and functional specialization

Planta

Ricardo A. Dante, Paolo A. Sabelli, Hong N. Nguyen, João T. Leiva-Neto, Yumin Tao, Keith S. Lowe, George J. Hoerster, William J. Gordon-Kamm, Rudolf Jung, Brian A. Larkins

B.A. Larkins
blarkins2@unl.edu; larkins@ag.arizona.edu
School of Plant Sciences, University of Arizona, 303 Forbes, Tucson, AZ 85721, USA

Electronic supplementary material

Online resource 1 RT-PCR primer sequences

Gene	Gene ID	Primer name	5' → 3' sequence
CYCA1;1	GRMZM2G017081	CYCA1;1-F	CCGTCTTTGGCAACAAGTGCTCGGAGTGTGAGTTC
		CYCA1;1-R	CACAGATGCGGTCAATTTCCATTGGGGCCACGG
CYCA1;2	GRMZM2G007113	CYCA1;2-F	GCGCGAAGTTGAATCCAGCTACCTCAGGTGC
		CYCA1;2-R	GCAACGAAGCCAGCATCGAGCAACCCCC
CYCB1;3	GRMZM2G005619	CYCB1;3-F	CGTGGATCCATGGAGTGCGCAAGGGAGTGGCGG
		CYCB1;3-R	CCGCTCGAGTCACTTCTTCTTCGGGGTGGTAATCC
CYCD2;1	GRMZM2G075117	CYCD2;1-F	CATCGCGGATCCGTGCCGGGCTATGACTGCGCCGCC
		CYCD2;1-R	ATCGCGGATCCTTAGAGTAGACGTCTAGTGATCCTT
CYCD5,1[a]	GRMZM2G006721	CYCD5;1-F	GCTGGATCATCAAGACCACGGCGATG
		CYCD5;1-R	GGCGACAGAGACGCCCGAGGACGC
CDKA;1	GRMZM2G008327	CDKA;1-F	GCGTACTGCCATTCTCATAGAGTTCTTCATCGAG
		CDKA;1-R	GGCAAACAACTGACTCCTGGCCAACTCTGC
CDKA;3	GRMZM2G174596	CDKA;3-F	ACCGCGACCTGAAGCCGCAGAACCTG
		CDKA;3-R	GGCGGCGCGGGCGGTGATCCTC
CDKB1;1	GRMZM2G495626	CDKB1;1-F	TTGCCCATCGACGGTAGACGAGAGCGAG
		CDKB1;1-R	AGTCACCAGGAAAGAGAGCCTGCCGTCG
ACT1	GRMZM2G126010	ACT1-F	ATTCAGGTGATGGTGTGAGCCACAC
		ACT1-R	GCCACCGATCCAGACACTGTACTTCC

[a] *CYCD5;1* primers match the *CYCD5;2* sequence perfectly. Because both genes are expressed in endosperm, their expression is not distinguished by RT-PCR. Hence, *CYCD5;1* and *CYCD5;2* are collectively referred to as CYCD5 in this work

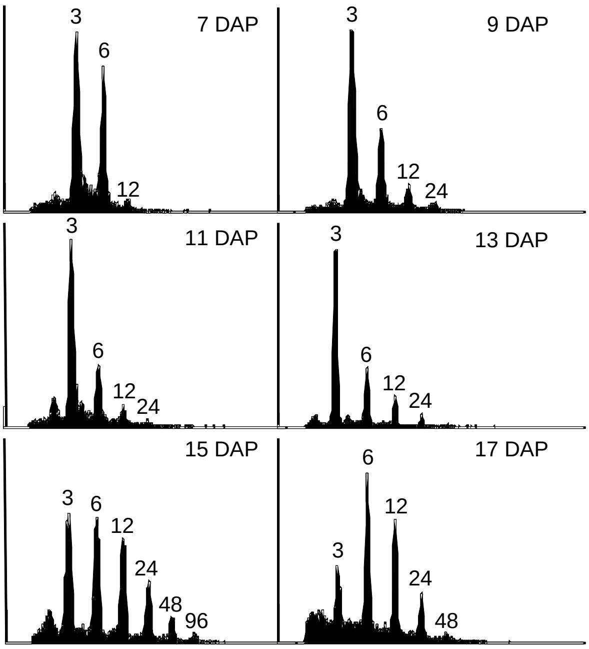

Online resource 2 Flow cytometric analysis of endosperm development. Endosperms from kernels at different DAP were manually dissected from pericarp, nucellus and embryo, and analyzed essentially as described by Dilkes et al. (2002). Briefly, three to five dissected endosperms were pooled and chopped with a razor blade in ice-cold PARTEC buffer (200 mM Tris-HCl pH 7.5, 4 mM $MgCl_2$, and 0.1% Triton X-100). The homogenate was sequentially filtered through cheesecloth and a 100-µm nylon mesh, stained with 4,6-diamidino-2-phenylindole (DAPI; Sigma) at 2 µg/ml, and analyzed with a CAII flow cytometer (PARTEC, Münster, Germany). A minimum of 10,000 nuclei per sample were counted and analyzed with the WinMDI Version 2.8 software (Joseph Trotter, The Scripps Research Institute, http://facs.scripps.edu/software.html). Representative assays are shown for each developmental stage. The abscissa and ordinate show fluorescence in logarithmical scale and number of nuclei, respectively. Numbers above peaks indicate their ploidy levels, expressed as C-values. At 7 DAP, 3C and 6C nuclei (indicative of cells in G1 and G2 phases of the mitotic cell cycle) are predominant, and endoreduplicated (>6C) nuclei are rare. Endoreduplicated nuclei progressively accumulate during the 9-13 DAP period. By 15 DAP, endosperm shows extensive endoreduplication, as a large proportion of nuclei are recruited into the >6C ploidy classes and ploidy values reach 96C. The decrease in frequency of highest-ploidy nuclei and increase of fluorescence signal at low values at 17 DAP are indicative of programmed cell death onset subsequent to endoreduplication

Reference

Dilkes BP, Dante RA, Coelho C, Larkins BA (2002) Genetic analyses of endoreduplication in *Zea mays* endosperm: evidence of sporophytic and zygotic maternal control. Genetics 160:1163-1177

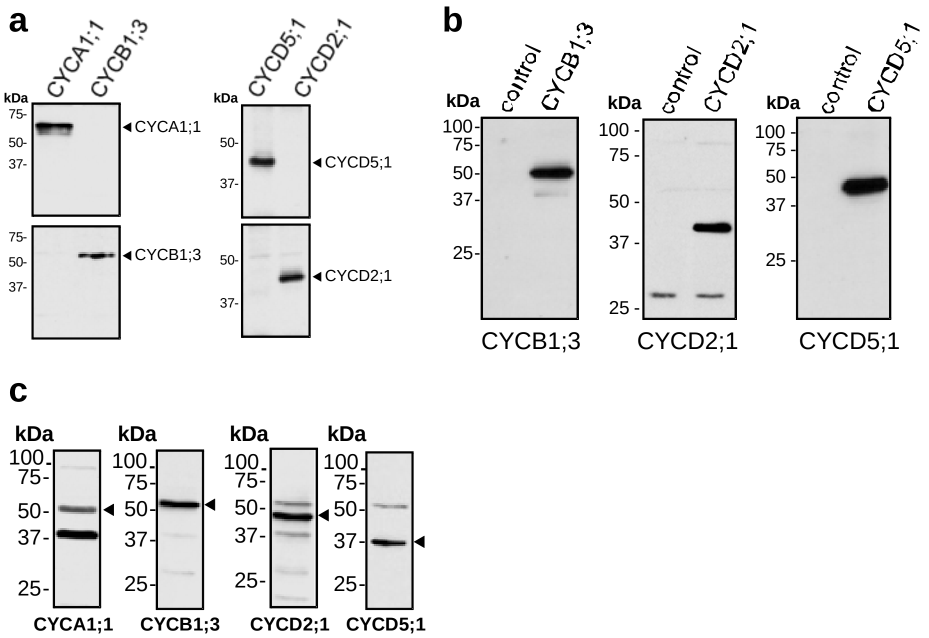

Online resource 3 Characterization of specific affinity-purified antibodies against maize cyclins. **a**, Immunoblot analysis of *in vitro* synthesized CYCA1;1 and CYCB1;3 with CYCA1;1, and CYCB1;3 antibodies, and *in vitro* synthesized CYCD5;1 and CYCD2;1 with CYCD5;1 and CYCD2;1 antibodies. One microliter of synthesis reactions was immunoblotted as described in Materials and Methods. Upper panels, immunoblots with CYCA1;1 or CYCD5;1 antibodies; lower panels, immunoblots with CYCB1;3 or CYCD2;1 antibodies. Protein positions are indicated by arrowheads. CYCA1;1 and CYCB1;3 antibodies, and CYCD5;1 and CYCD2;1 antibodies were raised against the N- and C-terminal regions of the corresponding proteins, respectively. Because these regions share limited sequence identity, the cross-reactivity between the antibodies raised against the N- and C-terminal regions was assessed. *In vitro* translated CYCA1;1 and CYCB1;3 proteins were specifically recognized by their corresponding antibodies in immunoblot analyses. CYCA1;1 and CYCB1;3 migrated on SDS-PAGE as polypeptides of approximately 58 kDa and 53 kDa, respectively, in accordance with their predicted molecular masses. *In vitro*-translated CYCD5;1 and CYCD2;1 proteins were specifically recognized by their corresponding antibodies in immunoblot analyses as approximately 45-kDa polypeptides. **b**, Immunoblot analysis of CYCB1;3, CYCD2;1 and CYCD5;1 expressed in S2 cells. Transfections were performed with control empty vectors or vectors containing coding regions of cyclins (indicated above panels) as described in Materials and Methods. Antibodies used for immunoblotting are indicated below panels. Polypeptides of apparent molecular masses similar to those produced *in vitro* were specifically recognized by CYCB1;3, CYCD5;1, and CYCD2;1 antibodies in extracts of S2 cells transfected with the corresponding constructs. CYCA1;1 was not expressed successfully in S2 cells. **c**, Immunoblot analysis of total soluble protein (50 μg per lane) from 9-DAP endosperm extracts with CYCA1;1, CYCB1;3, CYCD2;1 and CYCD5;1 antibodies, as indicated below panels. Cyclin proteins are indicated by arrowheads. All antibodies recognized polypeptides with an apparent molecular mass similar to that of the respective full-length *in vitro* translation products and S2 cell-expressed proteins. CYCA1;1

antibodies recognized a doublet of approximately 53 kDa. A major polypeptide of approximately 40 kDa and, to a lesser degree, an approximately 100-kDa polypeptide were also detected by CYCA1;1 antibodies. CYCB1;3 antibodies recognized a major polypeptide of approximately 53 kDa. CYCD5;1 antibodies recognized a major 37-kDa polypeptide in addition to a 55-kDa polypeptide and CYCD2;1 antibodies recognized a major polypeptide of approximately 48 kDa. In every case, these polypeptides were detected specifically by the affinity-purified antibodies and not by antibodies that were prepared similarly from their respective pre-immune sera (not shown). CYCD2;1 and CYCD5;1 molecular masses in immunoblots of developing endosperm were similar to those observed in immunoblots of germinating maize embryonic axes (Gutiérrez et al. 2005; Lara-Núñez et al. 2008)

References

Gutiérrez R, Quiroz-Figueroa F, Vázquez-Ramos JM (2005) Maize Cyclin D2 expression, associated kinase activity and effect of phytohormones during germination. Plant Cell Physiol 46:166-173

Lara-Núñez A, de Jesus N, Vázquez-Ramos JM (2008) Maize D4;1 and D5 cyclin proteins in germinating maize. Associated kinase activity and regulation by phytohormones Physiol Plant 132:79-88

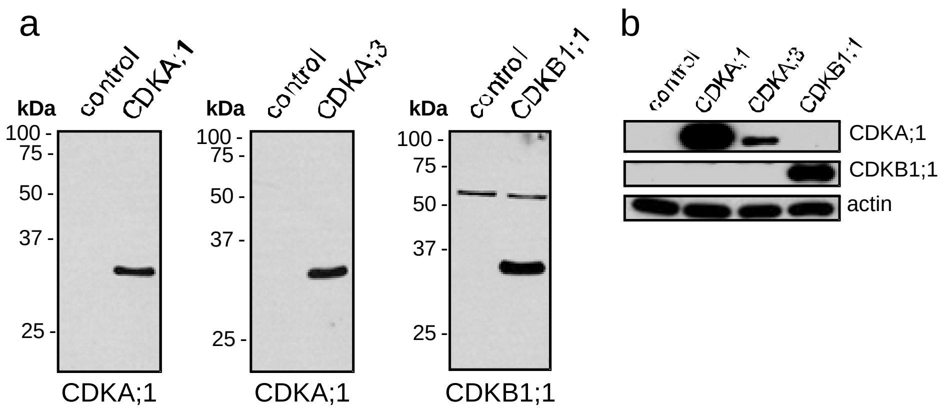

Online resource 4 Characterization of specific affinity-purified antibodies against maize CDKs. To characterize affinity-purified polyclonal antibodies generated against full-length CDKA;1 and CDKB1;1, these proteins were expressed in Drosophila S2 cells, and cell lysates were analyzed by immunoblotting as described in Materials and Methods. Transfected constructs are indicated above panels. **a**, Detection of A-type CDKs and CDKB1;1 by antibodies raised against CDKA;1 and CDKB1;1. Antibodies used in the immunoblots are indicated below panels. CDKs were recognized as polypeptides of approximately 34 kDa in extracts of S2 cells transfected with respective expression constructs, but not in cells transfected with control vector. CDKA;1 antibodies cross-reacted with CDKA;3 protein; consequently these antibodies could not distinguish between CDKA;1and CDKA;3 in maize cell extracts. **b**, Specificity of CDKA and CDKB1;1 antibodies. Antibodies used are indicated on the right. CDKA;1 antibodies did not recognize CDKB1;1. Conversely, CDKB1;1 antibodies recognized exclusively CDKB1;1, but not CDKA;1 or CDKA;3. Therefore, antibodies were obtained that recognize specifically A-type CDKs or CDKB1;1. An actin immunoblot is shown as the loading control

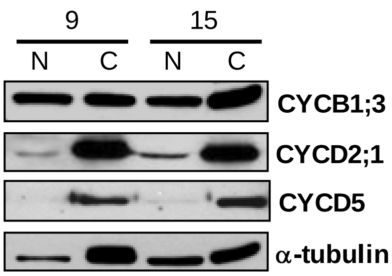

Online resource 5 Subcellular localization of cyclin proteins analyzed by immunohistochemistry in this work. Nuclear (N) and cytoplasmic (C) protein fractions were prepared from whole 9- and 15-DAP endosperms as described by Sabelli et al. (2005). Fractions (10 μg per lane) were immunoblotted with antibodies (indicated at right) against cyclins or α-tubulin. At 9 and 15 DAP, CYCB1;3 was localized to both nucleus and cytoplasm to similar degrees, while at 15 DAP its cytoplasmic localization was moderately increased in comparison with 9 DAP. At 9 and 15 DAP, CYCD2;1 was predominantly localized to cytoplasm, and its relative nuclear localization seemed to be enhanced at 15 DAP compared with 9 DAP. CYCD5 was almost exclusively localized to the cytoplasm at 9 and 15 DAP. α-tubulin was mostly cytoplasmic at both stages although by 15 DAP the nuclear component was accentuated. Thus, immunoblot analyses of cell fractions for individual cyclins were generally consistent with subcellular localization patterns that were computationally-predicted and observed through immunohistochemical analysis (Fig. 3). Previously, immunoblotting of endosperm cell fractions indicated that maize RBR1 and RBR3 proteins are localized to nuclei (Sabelli et al. 2005). The α-tubulin immunoblot shown here is reproduced from Sabelli et al. (2005), as the analyses of subcellular localization of RBR and cyclin proteins were part of common experiments.

Reference

Sabelli PA, Dante RA, Leiva-Neto JT, Jung R, Gordon-Kamm W, Larkins BA (2005) RBR3, a member of the retinoblastoma-related family from maize, is regulated by the RBR1/E2F pathway. Proc Natl Acad Sci USA 102:13005-13012
